# Computational analysis of dynamic allostery and control in the SARS-CoV-2 main protease

**DOI:** 10.1098/rsif.2020.0591

**Published:** 2021-01-06

**Authors:** Igors Dubanevics, Tom C. B. McLeish

**Affiliations:** 1 School of Natural Sciences, University of York, York, UK; 2 Department of Physics, University of York, York, UK

**Keywords:** allostery, protein dynamics, elastic network model, SARS-CoV-2

## Abstract

The COVID-19 pandemic caused by the novel coronavirus SARS-CoV-2 has no publicly available vaccine or antiviral drugs at the time of writing. An attractive coronavirus drug target is the main protease (M^pro^, also known as 3CL^pro^) because of its vital role in the viral cycle. A significant body of work has been focused on finding inhibitors which bind and block the active site of the main protease, but little has been done to address potential non-competitive inhibition, targeting regions other than the active site, partly because the fundamental biophysics of such allosteric control is still poorly understood. In this work, we construct an elastic network model (ENM) of the SARS-CoV-2 M^pro^ homodimer protein and analyse its dynamics and thermodynamics. We found a rich and heterogeneous dynamical structure, including allosterically correlated motions between the homodimeric protease's active sites. Exhaustive 1-point and 2-point mutation scans of the ENM and their effect on fluctuation free energies confirm previously experimentally identified bioactive residues, but also suggest several new candidate regions that are distant from the active site, yet control the protease function. Our results suggest new dynamically driven control regions as possible candidates for non-competitive inhibiting binding sites in the protease, which may assist the development of current fragment-based binding screens. The results also provide new insights into the active biophysical research field of protein fluctuation allostery and its underpinning dynamical structure.

## 1. Introduction

Over 2020, a rapidly spreading disease named COVID-19 caused by the novel coronavirus SARS-CoV-2 has generated a global pandemic. Preventive measures have been taken by a majority of countries, but no vaccine or anti-viral drugs exist, at the time of writing, although candidates are under trial. In the longer term therefore, the identification of all potential inhibitor sites at all points of the viral life cycle is of interest. Here we focus on the low-frequency dynamical structure of the virus’ main protease, an important molecular machine in the viral cycle, and identify critical residues in its allosteric control. The work is informative for inhibitor design by identifying control regions of the protein that are distant from, rather than proximal to, its active sites. Allosteric mechanisms for distant control of binding and activation fall into two main classes: those which invoke significant conformational change (the original scenario of Monod *et al*. [1]), and mechanisms that invoke the modification of thermal (entropic) fluctuations about a fixed, mean conformation [2–5]. Such ‘fluctuation allostery’ recruits mostly global, low-frequency modes of internal protein motion, which are well captured by correspondingly coarse-grained mechanical representations of the protein [6,7]. One effective tool at this level is the elastic network model (ENM) [8]. The ENM resolves protein structure at the level of alpha-carbon sites only, which are represented as nodes connected by harmonic springs within a fixed cut-off radius from each other. Local point mutation can be modelled by changing the moduli of springs attached to the corresponding residue, and effector binding by the addition of nodes (and local harmonic potentials) at the corresponding coordinates. The most significant contributions to both the correlated dynamics of distant residues, and to the entropy arising from structural fluctuation, come from global modes, whose ENM approximation allows straightforward calculation. This approach was successfully used to identify candidate control residues whose mutation may control allostery of effector binding in the homodimer transcription factor CAP [9].

This study, and others, have shown that, while the huge reduction in the number of degrees of freedom that the ENM constitutes does not capture the quantitative values of free energies, or the numerical changes to those values on mutation that are seen in experiment, it can rank them qualitatively, and also identify the functional form of the thermal dynamics of a protein for a significant set of low-frequency modes. Naturally, those aspects of protein function that rely on side-chain structures and interactions, or on very fast processes, are not captured by any coarse-grained model, including those of the ENM class. However, low-frequency dynamics and processes that modify them can be addressed by ENMs. In particular, the coarse-graining of the ENM can determine which residues present as candidates for allosteric control through mutation. The method, and the open software (DDPT) used in the previous study on allosteric homodimers, and confirmed by experimental calorimetry on model-designed mutations [10], is deployed here in a similar way (see Material and methods section) to a coarse-grained ENM of the SARS-CoV-2 main protease. The motivation for a numerical analysis of possible dynamically controlled allostery in this protein arises from recent experimental studies on the structurally similar SARS-3C-like protease that have already identified two regions where mutations inhibit the protein function without significant structural change [11,12]. This evidence that long-range dynamical effects are both implicated in, and offer control of, allosteric function in this family of proteins suggests a dynamic structure of sufficient richness to warrant a full analysis.

In the following, we present structural and functional detail of the SARS-CoV-2 M^pro^ as further essential background, then describe our simulations and their results, before concluding with a discussion of the potential to exploit its dynamical allostery for non-competitive inhibition.

### 1.1. The SARS-CoV-2 main protease protein

**Figure 1.**
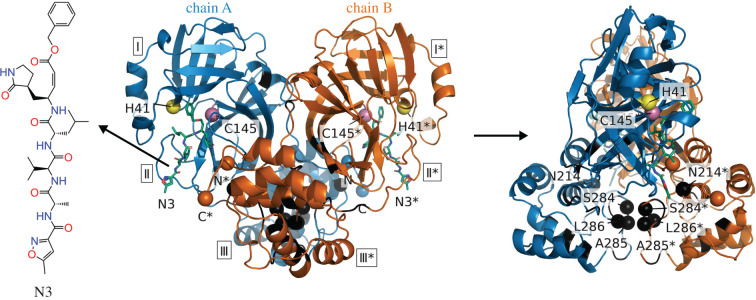
The crystal structure of the SARS-CoV-2 M^pro^ with N3 inhibitor. Chain A of the dimer is shown in blue, while chain B, in orange. Domains are labelled with boxed Roman numerals (I, II and III). Amino acid residues of the catalytic dyad are indicated as yellow spheres for H41 and magenta spheres for C145. Asterisks mark residues from chain B (orange). Chain termini are shown as spheres and labelled N and C for chain A (blue) and N* and C* for chain B (orange). The N3 inhibitor is shown as green sticks. Experimentally identified dynamically allosteric residues N214 and SAL284-286 are shown as labelled black spheres. Identified control residue candidates for dynamic allostery in the SARS-CoV-2 M^pro^ that are distant from the catalytically active residues H41 and C145 are coloured in black on both homodimeric chains. These residues can be found in table 1.

At the time of this study (14 December 2020) more than 71 000 papers have been published in relation to the virus (see COVID-19 Primer). However, work is still in progress to identify biological and molecular pathways the virus takes. Fortunately, significant research has already been directed to the very similar coronavirus—SARS-CoV, first identified in 2003. The SARS-CoV and SARS-CoV-2 genetic sequences are approximately 80% identical [13]. Both viruses encode a main protease (M^pro^), also known as the 3C-like protease (3CL${\;}^{\;pro}$). In its active form M^pro^ is a two-protomer homodimer with one active site per homodimer chain [14]. A recent crystal structure for the SARS-CoV-2 M^pro^ is shown in figure 1, alongside the co-crystallized N3 inhibitor, which occupies the active sites of the protease dimer, between domains II and III of each domain. Away from the dimer interface, the protein structure is dominated by beta-sheets, while the interface itself is composed of adjacent helices and loops. Although M^pro^ is a relatively compact protein (fewer than 310 residues per chain), it plays a vital role in the viral cycle of both coronaviruses: it divides polyproteins expressed from the viral mRNA into its functional non-structural units [15]. This functional role makes SARS-CoV-2 M^pro^ an appealing target for drug design. The major research effort to date has been focused on the competitive inhibition of SARS-CoV-2 M^pro^, i.e. by directly targeting the active site with molecules that competitively bind to the active site ‘pockets’ [13,16–18].

A significant body of work has been recently published investigating inhibitors for the SARS-CoV-2 main protease via virtual screening and molecular dynamics simulations [19–26]. In 2011, it was found that N214A mutation dynamically inactivates SARS-CoV M^pro^ [11]. The same research group later characterized another SARS-CoV M^pro^ mutation, S284-T285-I286/A, which dynamically enhances the protease catalytic activity more than threefold [12]. In SARS-CoV-2 M^pro^, two of those amino acids (T285 and I286) are changed to T285A and I286L with respect to SARS-CoV M^pro^. However, only a little enhancement in catalytic activity is observed [13]. This delicate potential control region will appear below in our analysis (see §2.2.2). Due to the high sequence conservation between the two coronaviruses, and 96% amino acid sequence identity between the main proteases, SARS-CoV-2 M^pro^ might possess similar allosteric features. Furthermore, in another study, researchers found the root mean square deviation (RMSD) of 0.53 Å for apo (ligand-free) forms of two corona viruses’ main proteases (PDB accession 2bx4 and 6y2e) [13]. These findings together evidence that the N214A mutation operates through a fluctuation allostery mechanism and structural similarities between two proteases motivate the analysis of the coarse-grained dynamic structure of SARS-CoV-2 M^pro^ reported here. In the following, we apply the ENM techniques of [9] to this purpose, looking in particular to identify non-active, yet allosteric, sites for non-competitive inhibition.

## 2. Simulations, results and discussions

No crystallographic structure of the SARS-CoV-2 M^pro^^1^ active form with a polyprotein is available to date, but only empty (apo) structures or structures with synthetic ligands/substrates attached to the active site. Therefore, the ENM study reported here used a recent crystallographic structure (PDB accession 6lu7 [16]) with competitively inhibited active site to calculate fluctuation free energies and consequent allosteric energies and their modification under mutation. The inhibited (with N3 inhibitor) and ligand-free structures of the protein are almost identical, shown in electronic supplementary material, figure S1. Resolutions for the structures shown are 2.16 Å and 1.75 Å, respectively, while RMSD between them is under 1.5 Å for C_*α*_ atoms. Evidently, very little structural change occurs upon inhibitor binding. These findings further support the hypothesis of dynamically driven allosteric control of SARS-CoV-2 M^pro^, and provide a structure (6lu7) on which to base an ENM construction (electronic supplementary material, Sec. A). All three PDB files used in this study were derived from original crystallographic structure of SARS-CoV-2 M^pro^ with N3 inhibitor.

**Figure 2.**
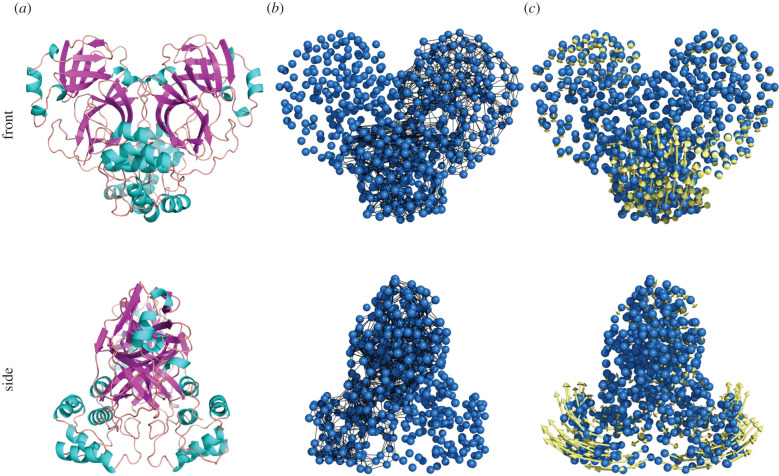
Constructing ENM of SARS-CoV-2 M^pro^ step-by-step. (*a*) SARS-CoV-2 M^pro^ secondary structure cartoon. (*b*) Elastic model of M^pro^ generated with PyANM package in PyMOL. C_*α*_ atoms are shown in blue; while node-connecting springs (black) are shown only for one chain for comparison. (*c*) The first real vibrational mode eigenvectors (yellow) visualization. For clarity, displacement vectors are scaled five times.

The resulting ENM of M^pro^ is shown in figure 2. It takes C_*α*_ node masses as the whole residue mass, and uses a cut-off distance for harmonic connecting springs of 8 Å, based on optimizing the comparison of mode structures between ENM and full molecular dynamics simulations, in previous work on catabolite activator protein [9,27] (electronic supplementary material, Sec. B). In the case of N3 inhibitor, short polypeptide AVL was coarse-grained in the same way as the main chain amino acids while the other heavy atoms (C, N and O) have been treated as individual nodes with atomic masses (electronic supplementary material, figure S2).

Balancing the requirements of: (i) sufficient spatial resolution of dynamics; (ii) requirements not to include unphysically small-scale structure; (iii) acceptable convergence of thermodynamic calculations; and (vi) compatibility with the previous studies [28] leads to the choice of summing the first real 25 modes in SARS-CoV-2 M^pro^ ENM calculations (electronic supplementary material, Sec. C). Although high-wavenumber modes may contain apparent contributions to allosteric effects, it is essential, when deploying ENM coarse-graining, to avoid including dynamic structures with length-scales comparable to the residue–residue spacing, where structural detail is no longer faithfully captured (for stability and convergence properties of free energies and allosteric response to mutations, see electronic supplementary material, section C, where the physically relevant low-frequency structure is differentiated from nonphysical contributions from high modes). We note that similar studies by others have employed as few as the first real 10 modes [29].

### 2.1. Residue–residue dynamic cross-correlation map

**Figure 3.**
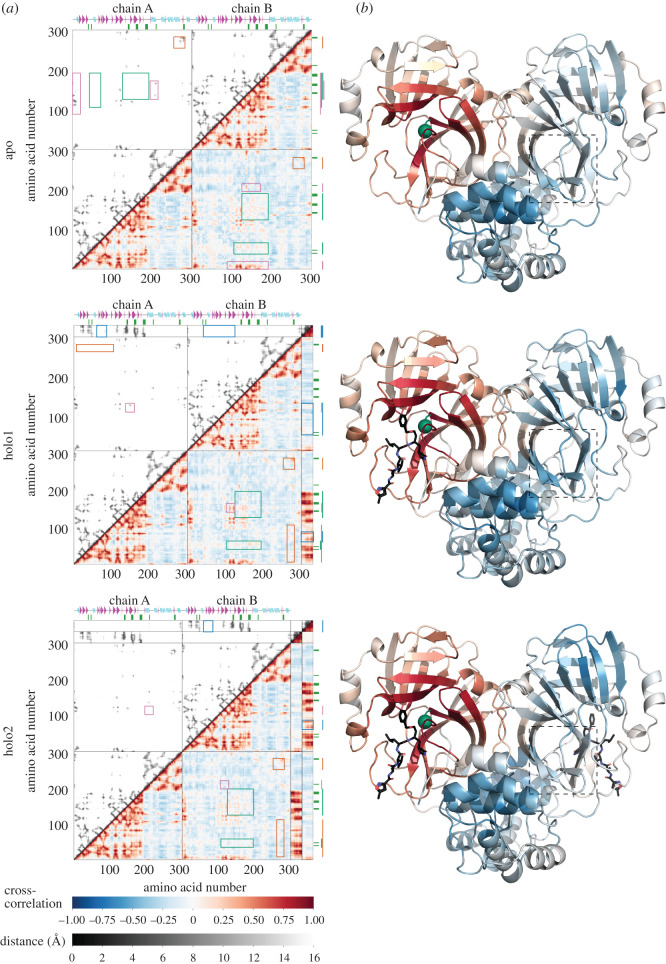
The cross correlation of the motion of 6lu7 ENM. (*a*) The cross-correlation maps calculated for the first real 25 modes (bottom-right region of the plots) and spacing between residues (C_*α*_ nodes) and ligand nodes (top-left region of the plots) for apo, holo1 and holo2 forms. The first colour scale shows the extent of cross correlation, with a cross correlation of 1 (red) indicating perfectly correlated motion, −1 (blue) showing perfectly anti-correlated motion and 0 (white) no correlation. The second colour scale (black to white) depicts the Euclidean distance between two ENM nodes in the Cartesian space in 0–16 Å range. The secondary structure of M^pro^ is indicated along the horizontal residue axes, with cyan waves indicating alpha helices, and magenta triangles indicating beta sheets. Coloured lines on the vertical axes point to the boxed regions on the plots mentioned in the main text. The green ticks on the axis indicate the location of the biologically active residues (table 1). (*b*) A real-space representation of the correlations in 6lu7 ENM with respect to residue 145 on chain A (green sphere). The dashed squares indicate the anti-correlation enhanced in apo–holo1 and reduced in holo1–holo2 transitions. The cross correlation matrix was calculated using only the C_*α*_ atoms for the protein and all heavy atoms for the ligand (N3 inhibitor).

The first quantity of interest is the map of dynamic correlations, which indicates for each residue on the protein those other residues whose motion correlates with its own (equation (4.1)). This gives a detailed summary map of the homodimer dynamical structure, and is also significant thermodynamically, since the same elastic communication drives both correlations and allosteric control [4]. The dynamic cross-correlation maps for all residues in the ENM apo (ligand-free), holo1 (only one active site at chain A occupied) and holo2 (both active sites occupied) structures are shown in figure 3. We discuss the dynamic features of each structure in the following.

**Table 1.** SARS-CoV-2 main protease key information used in this study: PDB ID; experimentally identified bioactive residues of SARS-CoV M^pro^ reported in the literature; active regions which we identified in this study, distant from the active site for SARS-CoV-2 M^pro^ (electronic supplementary material, Sec. D).

protein	PDB ID	experimentally identified bioactive residues	computationally identified distant control residues (this study)
SARS-CoV-2	6lu7	41, 49, 143-5, 163-7, 187-192 [20]	5, 9, 14, 109, 111, 127, 197-8, 200, 205, 214,
main protease		214, 284-6 [11,12]	264, 268-9, 272, 276-7, 281-2, 284-6, 292

#### 2.1.1. Apo

The N-terminus of each chain positively correlates with residues adjacent to the active site (res 100–200) on the other chain, reaching cross-correlation values up to 0.81. This is due to physical proximity rather than allostery (figure 3*a*, apo, wide purple rectangles in lower-right and upper-left quadrants). Significantly however, the dynamics of the regions around active sites on both chains positively correlate, in this case allosterically, with each other (figure 3*a*, apo, green square and rectangle in lower right quadrant). However, cross-correlation between the catalytic dyad residues on opposite chains is almost non-existent: 0.08 and 0.00 for H41 and C145, respectively, although H41–C145 correlation reaches a value of −0.18. The active site regions are spatially distant: they do not appear at the corresponding location on a proximity map (figure 3*a*, apo, green square and rectangle in upper left quadrant). The T201–N214 alpha helix (which contains the experimentally sensitive N214) on one chain dynamically anti-correlates with H41 on the opposite chain with values ranging from −0.11 to −0.40. The same helix, from residue 201 to 213, also anti-correlates with C145 on the opposite chain (from −0.10 to −0.32); while surprisingly (since its mutation is effective in allosteric control) N214 shows no correlation (−0.02) with the catalytically vital C145 at all. We observe strong positive correlation, however, between this helix and two regions forming the active site pocket: residues K137–N142 (loop) and E166–H172 (β-turn) with values reaching 0.50 (figure 3*a*, apo, narrow purple rectangles in lower right and upper left quadrants), although this correlation can partially be accounted for by spatial proximity. S284-L285-A286 residues (henceforth SLA) on one monomer show positive cross-chain dynamic coupling of motion with the identical residues on the other with cross-correlation value no lower than 0.42, in this case through spatial proximity (figure 3*a*, apo, orange squares in lower-right and upper-left quadrants), but a somewhat smaller positive correlation spans residues 275–306 (N terminus) on both chains with respect to SLA. This effect suggests strong SLA coupling to a large fraction of the protein domain not containing the active site. Furthermore, SLA positively correlates with the T201–N214 alpha helix (with maximum value of 0.20), which contains the experimentally determined dynamically allosteric residue N214 (0.14, 0.01 and 0.16 for each residue in the SLA, respectively). Finally, SLA negatively correlate with the active site's catalytic dyad (−0.23 and −0.25 on average for H41 and C145, respectively) and residues around it (figure 3*a*, apo, not shown). Thus, we observe both dynamic correlation at a distance, as well as that due to immediate spatial proximity, supporting previous findings regarding SARS-CoV M^pro^ dynamically allosteric inactivation.

#### 2.1.2. Holo1

The positively correlated dynamics between the active sites are strongly enhanced by addition of the first ligand, especially in regard to the two beta sheets (G146-I152 and V157-L167) and a beta turn between them, with most residue–residue cross-correlation values over 0.1 with 0.29 maximum (figure 3*a*, holo1, green square in the lower right quadrant). Also, we can clearly see an enhancement of negative cross-correlation in the real space representation around the active site on chain B (figure 3*b*, dashed squares in apo and holo1; electronic supplementary material, figure S3). Interestingly, and by contrast, the region displaying positive correlation around residues 50–70 with respect to residues around the active site in the apo form is decreased in the holo1 structure (figure 3*a*, holo1, green rectangle in the lower right quadrant). In the holo1 form, the structural symmetry of the apo form is broken, permitting an asymmetric correlation between chains A and B (figure 3, holo1, across the diagonal of the lower right quadrant). The biologically active residues (green ticks) show up in the ligand's correlation with (host) chain A. However, four other regions, not cited in the literature to date, also show strong dynamic correlation with the ligand. Two of them (res 17–32 and 120–131) can be vividly observed as spatially proximal to the ligand from the corresponding distance map. Two other regions exhibiting positive cross-correlation are, however, distant from the ligand (figure 3*a*, holo1, blue rectangles in lower right and upper left quadrants). These regions span residues 67–75 and 77–91, respectively, and include two beta sheets and a beta turn in each case with most of the values over 0.1 and a maximum value over 0.6. The previously reported dynamically allosteric residue 214 (chain B) correlates with the ligand on chain A (values ranging from −0.11 to 0.12); potentially due to spatial proximity. The closest ligand residue to N214 is 11.2 Å away. Moreover, the ligand shows positive correlation at distance with the beta sheets on the opposite chain (figure 3*a*, holo1, blue rectangles in upper right quadrant). The fact that the ligand's motion positively correlates with beta sheets at residues 67–75 and 77–91 on both chains suggests strong chain–ligand coupling to residues 67–91 on both homodimer chains. SLA and the residues around it dynamically couple to the same residues on the opposite chain (figure 3*a*, holo1, orange square in lower right quadrant). The structural *C*_2_ symmetry-breaking upon ligand binding decouples motion of residues which are distant from the active site (figure 3*a*, holo1, orange square in lower right quadrant). Thus these residues can engage in collective motion driven by spatial proximity to their neighbours. Not seen on apo cross-correlation map, the second half of the N261-N274 alpha helix on chain B appears to correlate positively with four distant residue groups in 15–100 region on chain A (figure 3*a*, holo1, orange rectangle in upper left and lower right quadrants). Moreover, addition of the ligand produced an interesting ‘H’ shape correlation (on the cross-correlation map) between four beta sheets, two on each chain: G146-I152 and V157-L167 on chain A, G109-Y118 and S121-R131 on chain B with values from −0.23 to 0.57 (figure 3*a*, holo1, purple square in lower right quadrant). This group motion across chains is partially caused by two neighbouring beta turns sticking out of active site protein domains (figure 3*a*, holo1, purple square in upper left quadrant).

#### 2.1.3. Holo2

When a second ligand is added, the strong correlation between the active sites present in holo1 is diminished (figure 3*a*, holo2, green square in lower right quadrant; figure 3*b*, dashed squares in holo1 and holo2), while previously lowered correlations elsewhere re-attain values in the apo form level (green rectangle in the same quadrant and figure). The region around SLA and the N261–N274 alpha helix, which shows strong dynamical coupling with the residue groups in interval 15–100 of the holo1 form, is reduced on binding the second effector to its previous apo level correlation (figure 3*a*, holo2, orange square and rectangle in lower right quadrant). Such loss (or equivalently, and in other cases, gain) of previous fluctuation correlations between distant sites, on binding at a third site, is the basis of mutation control of dynamical allostery [9]. The principal mechanism is the perturbation of the global eigenmode structure of the protein. A reduced correlation arises from a reduction, on binding, in the number of modes for which the two correlated sites are both near anti-nodes. This was recently identified in detail as a route to negative allostery [30]. The newly added ligand on chain B shows the same correlation-at-distance with the L66-A70 and V73-L75 beta sheets, excluding the beta turn in between them (figure 3*a*, holo1, blue rectangles in upper right quadrant). Nevertheless, the cross chain coupling between active site and the alpha helix (T201–N214) is further increased. The correlation is split into three distinctive regions in locally ‘wedge-like’ shapes on the map, two of which are around active site. These structures were also seen in the CAP cross-correlation map [9], and indicate the two contributing beta strands acting as a local hinge region. The third region is located around a beta sheet at S121-R131, which is not bound to the ligand (figure 3*a*, holo2, purple squares in lower right and upper left quadrants). The cross-correlation displays an interesting structure along the helix from residue N201 to T214: the third region is split into three zones: positive, negative and positive correlation. This sign change is reminiscent of the third harmonic of a standing wave with two nodal points (in three dimensions those are nodal planes). The negative correlation region is absorbed by the two positive regions as we reach residue N214. Unsurprisingly, the two ligands exhibit identical correlations with their host and opposite chains. Noteworthy, each ligand has dynamics correlating positively with the termini of the opposite chain, arising principally from the spatial proximity of C and N termini to the opposite chain's active site.

### 2.2. Mutation scans for thermodynamic control

**Figure 4.**
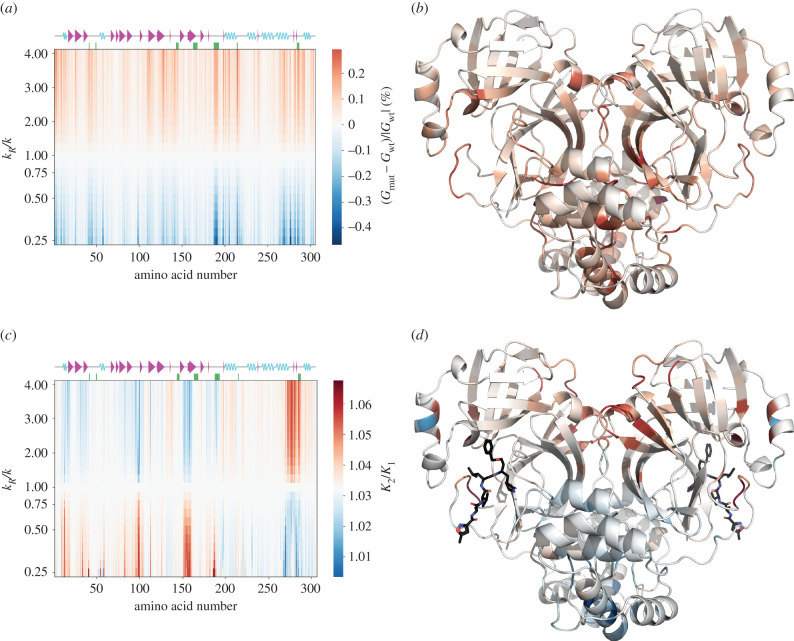
Mutation scan maps for thermodynamic control of M^pro^ calculated from the ENM over the first real 25 fluctuation modes. (*a*) A map for the fluctuation free energy change. The map plots the relative change in free energy to the wild-type ((*G*_mut_ − *G*_wt_)/|*G*_wt_|) due to the dimensionless change in the spring constant (*k*_*R*_/*k*) for the mutated residue with the amino acid number shown. White corresponds to values of free energy predicted by the wild-type ENM. Red corresponds to an increase in (*G*_mut_ − *G*_wt_)/|*G*_wt_| (decreased value of *G*_mut_ comparing to *G*_wt_), whereas blue corresponds to a decrease in (*G*_mut_ − *G*_wt_)/|*G*_wt_| (increased value of *G*_mut_ comparing to *G*_wt_). (*b*) The map for the vibrational free energy change plotted in real space onto the wild-type M^pro^ homodimer structure at *k*_*R*_/*k* = 4.00. (*c*) A map for the global control space of allostery in M^pro^. The map plots the change in cooperativity coefficient (*K*_2_/*K*_1_) due to the dimensionless change in the spring constant (*k*_*R*_/*k*) for the mutated residue with the amino acid number shown. White corresponds to values of *K*_2_/*K*_1_ predicted by the wild-type ENM. Red corresponds to an increase in *K*_2_/*K*_1_ (stronger negative cooperativity), whereas blue corresponds to a decrease in *K*_2_/*K*_1_ (weaker negative cooperativity or positive cooperativity). (*d*) The global map plotted in real space onto the wild-type M^pro^ homodimer structure at *k*_*R*_/*k* = 0.25.

The ENM calculations were extended to calculations of fluctuation-driven free energies (equation (4.2)) of various modelled wild-type and mutated state of SARS-CoV-2 M^pro^. ‘Point mutations’ are modelled in these ENM calculations, as in [9] by softening or stiffening all the harmonic springs attached to each residue in a complete scan of equally spaced spring moduli with ratios to their wild-type value in the range from 0.25 to 4.00. Previous work (by comparison to atomistic molecular dynamics and experimental calorimetry on mutations) has shown that this range is appropriately calibrated to the effects of mutations of a single step in side-change hydrophobicity [9]. Figure 4*a* reports the free energy changes (*G*_mut_ − *G*_wt_)/|*G*_wt_| induced in the entire homodimer when this scan of point mutations is made. Figure 4*c* reports the effect of the same mutational scan on the allosteric free energy *K*_2_/*K*_1_ (equation (4.3)) of binding between the two active sites.

#### 2.2.1. Free energy mutation scan on apo structure

All experimentally identified active sites (besides res 163–167) appear on the mutation scan of the 6lu7 apo structure, a somewhat remarkable result considering that no ligand is present to emphasize the special nature of the active sites. They appear dynamically predisposed to dynamic allosteric communication, in agreement with the cross-correlation map (figure 3). Both termini display mutation peaks due to their spatial proximity to the active sites. Additionally, a very sharp peak is seen around residues 187–192 where a free loop forming the active site is located. The seven new regions seen on the cross-correlation map (res 17–32, 67–75, 77–91, 97–98, 120–131, 201–214 and 261–274) form distinctive peaks on the mutation scan as well.

Furthermore, the experimentally identified residue N214 is signposted by these calculations: its computational mutation generates the largest fluctuation free energy change upon spring stiffening of 0.29% at *k*_*R*_/*k* = 4.00. The largest negative free energy change value of −0.47% is produced upon spring relaxation of M276 at *k*_*R*_/*k* = 0.25 (these two points define the amplitude of the colour bar of the figure). Although the catalytically paramount residue C145 is not as sharp as other peaks, it appears with greater strength in a higher mode summation (electronic supplementary material, figure S6A). The map is mostly qualitatively anti-symmetric around its mid-line (indicating the wild-type). However, the quantitative behaviour of three regions is worthy of attention: the region around residue 50, before 100, at residue 150, as well as the sharpest region around residues 187–192. Relaxation of stiffness at those points causes larger energy change than does stiffening. A very narrow region, not identified on the cross-correlation map, at residues 97–98, preceding a small beta sheet at res Y101–V104, appears sharply when local interaction strengths are relaxed. A new broad region of strong sensitivity to mutation appears on this map at residues 261–293, which includes an alpha helix at V261-N274. This helix is located on the surface of the protein far from the active site. This region also contains SLA which appear as sharp lines in figure 4*a*; and is especially responsive to spring constant change at L285: a free-energy change of 0.19% at *k*_*R*_/*k* = 4.00 and −0.29% at *k*_*R*_/*k* = 0.25. L285 is in the middle residue of the triad affecting two of its neighbours. Furthermore, L285 on one chain is in the closest contact with its counterpart on the other chain (5.3 Å).

We also note seven residues which are located on the homodimer chains’ interface (K5, P9, K12, E14, M276, I281 and S284), recalling that in the CAP homodimer, residues located on the interface were critical in allosteric regulation [9]. Especially responsive is E14 located on the very first alpha helix.

#### 2.2.2. Allosteric free energy mutation scan

The first result from the ENM calculation of the allosteric free energy for binding-site occupation is that *K*_2_/*K*_1_ = 1.032 ≈ 1 for the wild-type M^pro^. Therefore, this ENM (over 25 softest modes) is non-cooperative. Nevertheless, we can identify regions that are sensitive to even slight change in local stiffness which again are around biologically active areas. All previously marked active regions show up to some extent; especially vivid is the region around catalytic residue H41 and, as already appeared in the apo mutation scan, the loop around the active site (res 187–192). Residue N214 shows very weak allosteric control in this scan. The local environment around N214 is mainly hydrophobic (electronic supplementary material, figure S7). Therefore, the experimentally reported N214A mutation corresponds to a local structural stiffening (asparagine (N) is hydrophilic while alanine (A) is hydrophobic). This is indeed the region of parameter space (*k*_*R*_/*k* > 1) where this mutation displays a weak effect in the ENM, but a strong response upon relaxation. In SARS-CoV-2 M^pro^ residues T285 and I286 are replaced by L285 and A286 with respect to SARS-CoV M^pro^. Purely from the perspective of hydrophobicity of residue and environment, the former mutation would correspond to *k*_*R*_/*k* > 1, while the latter emulates *k*_*R*_/*k* < 1. However, no exact comparison with experimental data can be made as there are no data on how SLA simultaneous mutation affects SARS-CoV-2 M^pro^ catalytic activity. Without data on single mutations within the SLA region, we have no direct experimental verification of the single S284A mutation (which in our ENM corresponds to *k*_*R*_/*k* > 1 for similar reasons as for N214A mutation). We see a decrease in cooperativity for S284 spring stiffening, while for spring relaxation the ENM's cooperativity increases.

### 2.3. Two-point mutational scans

**Figure 5.**
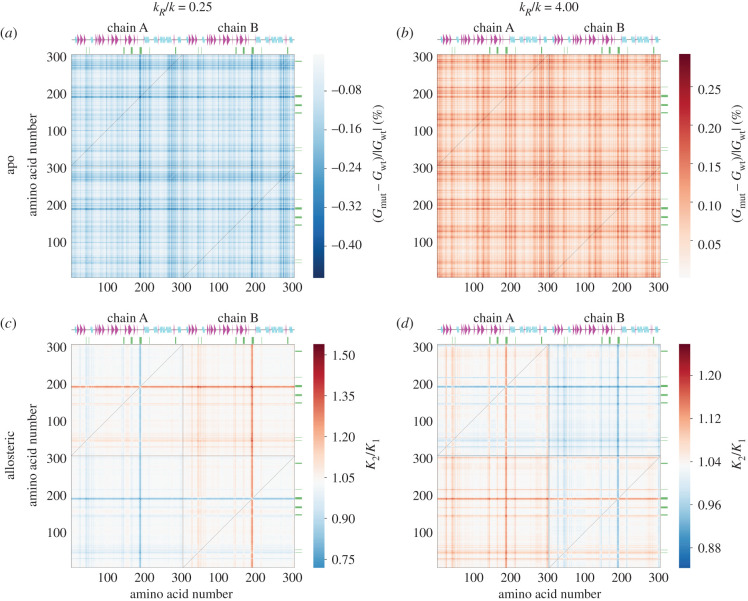
Two-point mutational maps for 6lu7 ENM with all possible pairwise combinations of residue mutations with equal spring constant change *k*_*R*_/*k* equal to 0.25 and 4.00 over the first real 25 fluctuation modes. (*a*,*b*) 2-point mutational maps for 6lu7 ENM with all possible pairwise combinations of residue mutations with equal spring constant change (*a*) *k*_*R*_/*k* = 4.00 (spring stiffening) and (*b*) *k*_*R*_/*k* = 0.25 (spring relaxation). (*c*,*d*) Maps for the two-dimensional global control space of allostery in M^pro^ for (*c*) *k*_*R*_/*k*=4.00 and (*d*) *k*_*R*_/*k* = 0.25 with colour bar centre at *K*_2_/*K*_1_ = 1.032. Black solid lines separate two homodimer chains, while dashed lines represent 1-point mutational scan results for the given spring constant change.

It is of interest to explore the cooperative effect of two-point mutations in models of fluctuation allostery, as previous work has indicated that double mutations may combine nonlinearly in control of the allosteric landscape of proteins [27]. This numerical scan explores cases where mutations are made on both or only on one of the single homodimeric chains: experimentally this is a possible, but not a trivial, task. However it can reveal contribution of each chain alone to fluctuation and allostery of the dimeric composite structure. The discussion of this section refers to results presented in the 2-point scans of 6lu7 ENM in figure 5. In order to present the response to all double-mutations on a single two-dimensional plot, the change in spring stiffness is not scanned; rather, just two constant spring changes of 0.25 and 4.00 are considered in a complete scan of residue pairs. Spring-stiffening 2-point mutational scans (figure 5*a*,*c*), *k*_*R*_/*k* = 4.00, model the effect of small molecule/ligand binding to the mutated residues (and would also model mutations such as N214A); while *k*_*R*_/*k* = 0.25 map looks at the opposite extreme to the stiffening case, which would model mutations that weaken local bonding (figure 5*b*,*d*). To explore possible nonlinearities in the combination of different mutation strengths, scans in the complementary parameter space of the two spring-constant changes were made for selected pairs of mutations (C145 and H41, already identified as important control sites). The corresponding two-dimensional plots of free energy and allosteric response are given in electronic supplementary material, figure S8.

#### 2.3.1. Free energy 2-point mutation scan on apo structure

The first measure, as in the single-point scans, is the difference in total free energy of the apo structure. As on the 1-point map for apo 6lu7 structure strong lines are observed (figure 5*a*,*b*), but spring relaxation resolves fewer biologically active residues than spring stiffening. In figure 5*a* only residues around H41, a loop region forming active site at D187-A191 and N214 show strong responses. On the other hand, stiffening (figure 5*b*) resolves all bioactive residues except H163-L167 (beta sheet forming the active site pocket) with an additional region around a small beta sheet (Y101-V104), alpha helices (T201-N214 and V261-N274) and loop region adjacent to the latter helix. As in the case of the 1-point map (figure 4*a*) M276 (−0.47%) and N214 (0.29%) define maximum absolute response upon relaxation and stiffening of these residues on both chains, respectively (figure 5*a*,*b*). In both cases, the SLA region shows moderate fluctuation free energy change. We conclude that stiffening is a better choice for resolving critical residues in fluctuation free energy control. Note that, in the case of this protein, the 2-point mutations combine approximately linearly: the effect of the first mutation (vertical lines) is not strongly affected by the second mutation. Nevertheless, responses to relaxation and stiffening give qualitatively different plots. The weak nonlinearities that are present are more evident when mutations on non-identical sites are combined (see electronic supplementary material, figure S8).

#### 2.3.2. Allosteric free energy 2-point mutation scan

Finally, the effect of all double mutations upon spring relaxation and stiffening on the allosteric free energy between the two active sites was calculated (figure 5*c*,*d*). While the 2-point apo maps show qualitatively different behaviour for relaxation and stiffening, the allosteric free energy 2-point mutation maps are qualitatively identical, modulo an inverted allosteric free energy change sign. A new strong control site that did not appear on the 2-point apo maps (figure 5*a*,*b*) is found at the beginning of the second beta-sheet (T25-L32) on each chain (figure 5*c*,*d*). Additionally, S1 and G302 exhibit strong allosteric control due to spatial proximity to the active site and, thus, the ligand. The apparent increased cooperativity for both mutations (in most cases) on chain A, and the same pattern of decreased cooperativity on chain B, is due to the formal symmetry breaking through choice of the first binding site at chain A. Mutation of residues around H41 constitutes an exception, and has an opposite effect on allostery to all others. The dashed lines in figure 5 represent the 1-point allo scan in figure 4 for corresponding *k*_*R*_/*k* values. In that scan *K*_2_/*K*_1_ values range from 1.003 to 1.068, while for these 2-point mutational scans the range is significantly increased: 0.719–1.540 for *k*_*R*_/*k* = 0.25 and 0.842–1.259 for *k*_*R*_/*k* = 4.00.

This calculation draws attention to an additional advantage of the 2-point scans: while N214 did not exhibit allosteric control on the 1-point map (figure 4*c*) over 25 fluctuation modes, it appears as a strong line in all quadrants on the 2-point maps. However, when the lines intersect in the cross-chain quadrant *K*_2_/*K*_1_ reaches almost wild-type value (corresponding to making exactly the same change on both monomers). This effect explains why we do not observe allosteric control of N214 on the 1-point mutational map: evidently, when the bold lines intersect (identical mutation or binding on both domains) allosteric effects interfere destructively. Thus, in the 1-point allo scan for 6lu7 ENM amplitude of *K*_2_/*K*_1_ is lower than in the 2-point mutational allosteric scan or hardly applies. The SLA region appears neither on the 1-point nor 2-point global maps for dynamic regulation of allostery in spite of its presence on the free-energy apo scans. This absence of control indicates the limited coupling between SLA and the given ligand (N3 inhibitor).

## 3. Discussion: what does ENM tell us about SARS-CoV-2 main protease?

The ENM analysis reinforces previous findings in application to other proteins, that in the SARS-CoV-2 M^pro^ as well, local harmonic potentials within the equilibrium protein structure, but without mean structural change, can identify already biologically active sites known from experiment. Furthermore, there is no need to possess holo forms of the protein to locate those active sites, whose correlated dynamics are already clear in the apo form. Calculations of those sites where total free energies are sensitive to mutations converge well with the limit of the sum over normal modes. The convergence of calculations of control of the allosteric free energy itself is more subject to noise, being a higher-order difference-quantity, but sufficient to identify strong candidates for control regions (electronic supplementary material, figure S6).

The analysis shows that SARS-CoV-2 M^pro^ possesses a rich dynamical structure that supports several long-distance allosteric effects through thermal excitation of global normal modes. In particular, the motions in the vicinity of two active sites are correlated within the first 25 non-trivial normal modes, especially in the singly bound dimer. Although, at the level of ENM calculations, this does not lead to cooperativity in the wild-type structure, it does render the protein susceptible to the introduction of cooperativity by mutation.

Our methodology is further supported by the ENM dynamics sensitivity to residue 214 and 284–286 mutations which have been shown by experiment to dynamically control SARS-CoV M^pro^.

The ENM calculations have identified new sites whose local thermal dynamics dynamically correlate with those of the active sites, and which also appear on global maps for allosteric control by single or double mutations. The new candidate control regions are summarized in table 1. In particular, residues around the beta sheets (Y101-V104, G109-Y118 and S121-R131) and the alpha helices (T201-N214 and V261-N274) are novel, and distant from the active site (figure 1). The positions of these residues suggest them as possible candidates for non-competitive inhibiting binding sites. We also draw attention to eight residues located on M^pro^ interface surface (table 1) as a further set of potential dynamically allosteric control residues.

Computational studies such as this, therefore, accompany and support concurrent experimental programmes of scanning for small-molecule binding candidates to the protein. We note that several candidate molecules, identified in very recent large crystallographic fragment screens against SARS-CoV-2 M^pro^ [31,32], bind to regions suggested as dynamically sensitive control candidates in this study. Such experimental programmes are essential in identifying inhibitors that are both effective, and have sufficient total binding free energies to the protein. Coarse-grained simulations such as the ENM method we have employed, while powerful in locating candidates for susceptible binding sites, cannot deliver predicted values for total free energies of binding. Fully atomistic computations are potential alternatives to experiment, as a recent study has shown for a (competitive) inhibitor for SARS-CoV-2 M^pro^, Leucoefdin (−41.71 kcal mol^−1^) [33], and nine other candidate ligands (with binding energies up to −82 kcal mol^−1^) [34]. However, to date there have been no calculations nor experiments on the binding energies of non-competitive yet inhibiting ligands. Because ENM calculations identify entropic contributions to free energy arising from fluctuations around a mean conformation, they typically deliver a sizeable fraction of the thermal energy *k*_B_*T* per mode affected. This sets the typical scale at a few kilojoules per mole for the free-energy changes predicted by the model, represented in the results presented here. Previous work has found that experimental free energies are typically ordered in the same sequence as the ENM values, though larger by factors that may arise through coupling of the global modes to unresolved local modes [4,9,35].

Furthermore, the ENM employed in this study was specific for the given inhibitor. Other ligands might, of course, show different behaviour in the corresponding holo structures and display other ‘hot-spots’; however, the appearance of active regions, and their coupling, in the apo structure suggests that there are general properties that emerge from the global elastic structure of the protein.

As well as providing specific information on the SARS-CoV-2 M^pro^ structure of the calculations reported there, the findings of this study also contribute to the large programme of research on fluctuation-induced allostery without conformational change. In particular, the general question of the focusing of dynamic correlations between distant (so candidate allosteric) sites is solved in a highly specific way by this structure. It also constitutes a system for which double mutations contribute in a predominantly linear, or weakly nonlinear, addition, in contrast to findings with other allosteric homodimers. This pattern includes the phenomenon we identified in the case of some single point mutations made identically and simultaneously in both monomers, whose cancellation in the 1-point mutational scans can mask their potential sensitivity as target sites. Finally, the appearance of control regions on the exterior surface of proteins, with obvious pharmacological application, generates other general questions in the biophysics of fluctuation elasticity in globular proteins.

## 4. Material and methods

Normal mode analysis (NMA) of ENM describes protein motions around equilibrium and can be used to calculate the partition function for large-scale harmonic thermal fluctuations in protein structure, including those responsible for allostery [36]. Two main approximations of NMA are:

— The structure fluctuates about a local energy minimum. Consequently, no other structures beyond the given equilibrium can be explored.
— The force field everywhere arises from sums over ENM harmonic.

The whole NMA method can be reduced to three steps:

1. Construct mass-weighted Hessian for a system. For a protein ENM the system consists of the coordinates of the C-alpha atoms (*N*) for each residue from the corresponding PDB structure.
2. Diagonalize the mass-weighted Hessian to find eigenvectors and eigenvalues of the normal modes.
3. Calculate the partition function (and so free energy) from the product over the normal mode harmonic oscillations.

The diagonalization of the 3*N* × 3*N* mass-weighted Hessian matrix is written as

$$A^{-1}\tilde{H}\;A=\Lambda ,$$

where ${\tilde{H}}_{ij}=\partial^{2}V_{ij}/\partial r_{i}\sqrt{m_{i}}\partial r_{\;j}\sqrt{m_{\;j}}$: the potential energy function *V*; distance between nodes *r*; node masses *m*. The eigenvectors of the mass-weighted Hessian matrix, columns of **A**, are the normal mode eigenvectors **a**:

$$A=\left(\begin{array}{cccc} | & | & & |\\ a_{1} & a_{2} & \cdots & a_{3N}\\ | & | & & | \end{array}\right).$$

***Λ*** is a 3*N* × 3*N* diagonal matrix with diagonal values equal to the associated normal modes’ squared angular frequencies *ω*^2^. The potential function used in this study is

$$V_{ij}=\left\{ \begin{array}{cc} \frac{k_{ij}}{2}{\left(r_{ij}-r_{ij}^{\left(0\right)}\right)}^{2}\; & r_{ij}^{2}\leq r_{c}^{2}\;\\ 0\; & r_{ij}^{2}>r_{c}^{2},\; \end{array}\right.$$

where *r*_*c*_ is a cut-off radius, which for this work is set at 8 Å; while *r*^(0)^ is the equilibrium distance between nodes derived from PDB crystallographic structure. For the wild-type protein, all spring constants are equal: *k*_*ij*_ = *k* = 1 kcal Å^−2^ mol^−1^.

### 4.1. Cross-correlation of motion

The cross-correlation *C* is estimated between an ENM node pair as a normalized dot product sum between their normal mode eigenvectors over *v* modes:

4.1 $$C_{ij}=\sum_{v}\left(\frac{a_{i}(v)\cdot a_{\;j}(v)}{\sqrt{|a_{i}(v){|}^{2}|a_{\;j}(v){|}^{2}}}\right).$$

*C* value of 1 implies perfectly correlated motion, −1 perfectly anti-correlated motion and 0 implies totally non-correlated motion.

### 4.2. Normal mode fluctuation free energy

Using statistical mechanics, it is possible to calculate an estimate to the fluctuation free energy of a system using the frequency of vibrations such as the normal modes. For this method, the partition function for the quantum harmonic oscillator [37], *Z*, for normal mode *k* is given as

$$Z_{k}=\frac{\exp\left(-(1/2)(\hbar \omega_{k}/k_{B}T)\right)}{1-\exp\left(-\hbar \omega_{k}/k_{B}T\right)},$$

where *k*_B_ is Boltzmann's constant, ℏ is the reduced Planck's constant, *T* is temperature in kelvin and *ω* is, already mentioned, angular frequency. Gibbs free energy (for a given mode) expressed in terms of partition function, with an approximation of little change in volume, can be written as

4.2 $$G_{k}=-k_{B}T\;\ln\left(\frac{1}{1-\exp\left(-\hbar \omega_{k}/k_{B}T\right)}\right)+\frac{1}{2}\hbar \omega_{k}.$$

### 4.3. Ligand dissociation constant

When free energy change Δ*G* (electronic supplementary material, Sec. C) is known for a dissociation reaction, corresponding dissociation constant *K* can be estimated via

4.3 $$K=\exp\left(-\frac{\Delta G}{k_{B}T}\right).$$

## Supplementary Material

6lu7_front.pse

## Supplementary Material

6lu7_side.pse

## Supplementary Material

6lu7-0.pdb

## Supplementary Material

6lu7-1.pdb

## Supplementary Material

6lu7-2.pdb

## Supplementary Material

6lu7-1point-25-Grel-0.xlsx

## Supplementary Material

6lu7-1point-25-K2K1.xlsx

## Supplementary Material

6lu7-2point-25-Grel-0-0.25.xlsx

## Supplementary Material

6lu7-2point-25-Grel-0-4.00.xlsx

## Supplementary Material

6lu7-2point-25-K2K1-0.25.xlsx

## Supplementary Material

6lu7-2point-25-K2K1-4.00.xlsx

## Supplementary Material

6lu7-croscor-25-0.xlsx

## Supplementary Material

text-SI.pdf

## Supplementary Material

6lu7-croscor-25-1.xlsx

## Supplementary Material

6lu7-croscor-25-2.xlsx

## Supplementary Material

6lu7-croscor-25-2.xlsx

## Supplementary Material

6lu7-dist-1.xlsx

## Supplementary Material

6lu7-dist-2.xlsx

## Supplementary Material

README.md

## Supplementary Material

6lu7-wt-G.xlsx

## Footnotes

1 From now on, M^pro^ will refer to the SARS-CoV-2 main protease protein.

## Data accessibility

All processed data are included in the paper and electronic supplementary material. Sufficient information and data are provided in and with the paper to allow others to replicate all study findings reported in this research article. DDPT source code can be accessed at https://sourceforge.net/projects/durham-ddpt.

## References

[1] Monod J, Wyman J, Changeux JP 1965 On the nature of allosteric transitions—a plausible model. J. Mol. Biol. 12, 88–118. (10.1016/S0022-2836(65)80285-6) 14343300

[2] Cooper A, Dryden DT 1984 Allostery without conformational change. A plausible model. Eur. Biophys. J. 11, 103–109. (10.1007/BF00276625) 6544679

[3] Hawkins RJ, McLeish TC 2006 Dynamic allostery of protein alpha helical coiled-coils. J. R. Soc. Interface 3, 125–138. (10.1098/rsif.2005.0068) 16849225 PMC1618481

[4] McLeish TCB, Rodgers TL, Wilson MR 2013 Allostery without conformation change: modelling protein dynamics at multiple scales. Phys. Biol. 10, 056004 (10.1088/1478-3975/10/5/056004) 24021665

[5] Schaefer C, VonDerHeydt AC, McLeish T 2018 The ‘allosteron’ model for entropic allostery of self-assembly. Phil. Trans. R. Soc. B 373, 20170186 (10.1098/rstb.2017.0186) 29735739 PMC5941180

[6] Go N, Noguti T, Nishikawa T 1983 Dynamics of a small globular protein in terms of low-frequency vibrational modes. Proc. Natl Acad. Sci. USA 80, 3696–3700. (10.1073/pnas.80.12.3696) 6574507 PMC394117

[7] Brooks B, Karplus M 1985 Normal modes for specific motions of macromolecules: application to the hinge-bending mode of lysozyme. Proc. Natl Acad. Sci. USA 82, 4995–4999. (10.1073/pnas.82.15.4995) 3860838 PMC390485

[8] Bahar I, Atilgan A, Erman B 1997 Direct evaluation of thermal fluctuations in proteins using a single-parameter harmonic potential. Fold. Des. 2, 173–181. (10.1016/S1359-0278(97)00024-2) 9218955

[9] Rodgers TL, Townsend PD, Burnell D, Jones ML, Richards SA, McLeish TC, Pohl E, Wilson MR, Cann MJ 2013 Modulation of global low-frequency motions underlies allosteric regulation: demonstration in CRP/FNR family transcription factors. PLoS Biol. 11, e1001651 (10.1371/journal.pbio.1001651) 24058293 PMC3769225

[10] Rodgers TL, Burnell D, Townsend PD, Pohl E, Cann MJ, Wilson MR, McLeish TC 2013 DDPT: a comprehensive toolbox for the analysis of protein motion. BMC Bioinf. 14, 183 (10.1186/1471-2105-14-183) PMC3689072 23758746

[11] Shi J, Han N, Lim L, Lua S, Sivaraman J, Wang L, Mu Y, Song J 2011 Dynamically-driven inactivation of the catalytic machinery of the SARS 3C-like protease by the N214A mutation on the extra domain. PLoS Comput. Biol. 7, e1001084 (10.1371/journal.pcbi.1001084) 21390281 PMC3044768

[12] Lim L, Shi J, Mu Y, Song J 2014 Dynamically-driven enhancement of the catalytic machinery of the SARS 3C-like protease by the S284-T285-I286/A mutations on the extra domain. PLoS ONE 9, e101941 (10.1371/journal.pone.0101941) 25036652 PMC4103764

[13] Zhang L, Lin D, Sun X, Curth U, Drosten C, Sauerhering L, Becker S, Rox K, Hilgenfeld R 2020 Crystal structure of SARS-CoV-2 main protease provides a basis for design of improved *α*-ketoamide inhibitors. Science 368, 409–412. (10.1126/science.abb3405) 32198291 PMC7164518

[14] Muramatsu T, Takemoto C, Kim YT, Wang H, Nishii W, Terada T, Shirouzu M, Yokoyama S 2016 SARS-CoV 3CL protease cleaves its C-terminal autoprocessing site by novel subsite cooperativity. Proc. Natl Acad. Sci. USA 113, 12 997–13 002. (10.1073/pnas.1601327113) 27799534 PMC5135343

[15] Pillaiyar T, Manickam M, Namasivayam V, Hayashi Y, Jung SH 2016 An overview of severe acute respiratory syndrome–coronavirus (SARS-CoV) 3CL protease inhibitors: peptidomimetics and small molecule chemotherapy. J. Med. Chem. 59, 6595–6628. (10.1021/acs.jmedchem.5b01461) 26878082 PMC7075650

[16] Jin Z *et al* 2020 Structure of M^*pro*^ from SARS-CoV-2 and discovery of its inhibitors. Nature 582, 289–293. (10.1038/s41586-020-2223-y) 32272481

[17] Dai W *et al* 2020 Structure-based design of antiviral drug candidates targeting the SARS-CoV-2 main protease. Science 368, 1331–1335. (10.1126/science.abb4489) 32321856 PMC7179937

[18] Jin Z *et al* 2020 Structural basis for the inhibition of SARS-CoV-2 main protease by antineoplastic drug Carmofur. Nat. Struct. Mol. Biol. 27, 529–532. (10.1038/s41594-020-0440-6). 32382072

[19] Di Paola L, Giuliani A 2020 Mapping active allosteric loci SARS-CoV spike proteins by means of protein contact networks. (http://arxiv.org/abs/200305200).

[20] Chen YW, Yiu CPB, Wong KY 2020 Prediction of the SARS-CoV-2 (2019-nCoV) 3C-like protease (3CL pro) structure: virtual screening reveals velpatasvir, ledipasvir, and other drug repurposing candidates. F1000Research 9, 129 (10.12688/f1000research.22457.2) 32194944 PMC7062204

[21] Ou X *et al.* 2020 Characterization of spike glycoprotein of SARS-CoV-2 on virus entry and its immune cross-reactivity with SARS-CoV. Nat. Commun. 11, 1620 (10.1038/s41467-020-15562-9) PMC7100515 32221306

[22] Ton AT, Gentile F, Hsing M, Ban F, Cherkasov A 2020 Rapid identification of potential inhibitors of SARS-CoV-2 main protease by deep docking of 1.3 billion compounds. Mol. Inform. 39, e2000028 (10.1002/minf.202000028) 32162456 PMC7228259

[23] Kandeel M, Al-Nazawi M 2020 Virtual screening and repurposing of FDA approved drugs against COVID-19 main protease. Life Sci. 251, 117627 (10.1016/j.lfs.2020.117627) 32251634 PMC7194560

[24] Estrada E 2020 Topological analysis of SARS CoV-2 main protease. bioRxiv 30, 061102. 10.1063/5.0013029 PMC7286701 32611087

[25] Hofmarcher M *et al.* 2020 Large-scale ligand-based virtual screening for SARS-CoV-2 inhibitors using deep neural networks. *SSRN Electron. J* (10.2139/ssrn.3561442)

[26] Elmezayen AD, Al-Obaidi A, Şahin AT, Yelekçi K 2020 Drug repurposing for coronavirus (COVID-19): in silico screening of known drugs against coronavirus 3CL hydrolase and protease enzymes. J. Biomol. Struct. Dyn. (10.1080/07391102.2020.1758791). PMC7189413 32306862

[27] Townsend PD *et al.* 2015 The role of protein-ligand contacts in allosteric regulation of the *Escherichia coli* catabolite activator protein. J. Biol. Chem. 290, 22 225–22 235. (10.1074/jbc.M115.669267) PMC4571973 26187469

[28] Townsend PD, Rodgers TL, Pohl E, Wilson MR, McLeish TC, Cann MJ 2015 Global low-frequency motions in protein allostery: CAP as a model system. Biophys. Rev. 7, 175–182. (10.1007/s12551-015-0163-9) 26000062 PMC4432019

[29] Guarnera E, Berezovsky IN 2016 Structure-based statistical mechanical model accounts for the causality and energetics of allosteric communication. PLoS Comput. Biol. 12, e1004678 (10.1371/journal.pcbi.1004678) 26939022 PMC4777440

[30] McLeish TCB, VonDerHeydt AC 2019 How proteins’ negative cooperativity emerges from entropic optimisation of versatile collective fluctuations. J. Chem. Phys. 151, 215101 (10.1063/1.5123741) 31822099

[31] Diamond Synchrotron. 2020 Main protease structure and XChem fragment screen. See https://www.diamond.ac.uk/covid-19/for-scientists/Main-protease-structure-and-XChem.html. (accessed 18 October 2020)

[32] El-Baba TJ *et al.* 2020 Allosteric inhibition of the SARS-CoV-2 main protease: insights from mass spectrometry-based assays. Angew. Chem. Int. Ed. 59, 23 544–23 548. (10.1002/anie.202010316) PMC7461284 32841477

[33] Singh A, Mishra A 2020 Leucoefdin a potential inhibitor against SARS CoV-2 Mpro. J. Biomol. Struct. Dyn. (10.1080/07391102.2020.1777903). PMC7309301 34281489

[34] Choudhary MI, Shaikh M 2020 In silico identification of potential inhibitors of key SARS-CoV-2 3CL hydrolase (Mpro) via molecular docking, MMGBSA predictive binding energy calculations, and molecular dynamics simulation. PLoS ONE 15, e0235030 (10.1371/journal.pone.0235030) 32706783 PMC7380638

[35] Hawkins RJ, McLeish TC 2006 Coupling of global and local vibrational modes in dynamic allostery of proteins. Biophys. J. 91, 2055–2062. (10.1529/biophysj.106.082180) 16798805 PMC1557547

[36] Bahar I, Lezon TR, Bakan A, Shrivastava IH 2010 Normal mode analysis of biomolecular structures: functional mechanisms of membrane proteins. Chem. Rev. 110, 1463–1497. (10.1021/cr900095e) 19785456 PMC2836427

[37] Blundell SJ, Blundell KM 2009 Concepts in thermal physics. Oxford, UK: Oxford University Press.

