# Supplementary material for: Computational analysis of dynamic allostery and control in the SARS-CoV-2 main protease
Source: J R Soc Interface. 2021 Jan 6;18(174):20200591. doi: 10.1098/rsif.2020.0591 (PMC7879766; doi:10.1098/rsif.2020.0591)
Supplement: text-SI.pdf [file rsif20200591supp13.pdf]

Supplementary Information for

Computational Analysis of Dynamic Allostery and Control in the SARS-CoV-2 Main Protease

Igors Dubanevics and Tom C.B. McLeish

Corresponding author: Tom C.B. McLeish
E-mail: tom.mcleish@york.ac.uk

Journal: Journal of the Royal Society Interface

This PDF file includes:

Supplementary text
Figs. S1 to S8
SI References

Supporting Information Text

A. PDB structures. Atoms of bound molecules were manually removed from the original 6lu7 PDB file to make up the three protein forms: ligand-free (apo); singly-bound ligand to chain A (holo1); each chain, A and B, possess one ligand (holo2). PDB files used in this study are attached as Supporting Information Files.

B. DDPT. This section enumerates the routine used to generate and analyse M^{pro} (6lu7) ENM via the Durham Dynamic Protein Toolbox (DDPT) (1). As an example, the routine is carried out explicitly here for the 6lu7 holo1 form.
First, the ENM interaction matrix is generated using the GENENMM function in DDPT:

GENENMM -pdb 6lu7_holo1.pdb -c 8 -ca -het -mass -res -fcust ffile

The crystallographic structure of a protein is assigned by `-pdb` flag. The cut-off distance (`-c`, by default 12 Å) was set to 8 Å and heteroatoms (`HETATM` in PDB notation) from the PDB file were included using `-het`, if required. Hookean spring strengths (by default 1 kcal $Å^{-2}$ mol^{-1}) around selected residues can be altered via the `-fcust` flag. The associated `ffile` specifies which residue springs' moduli are modified. By default, all ENM nodes' masses (atoms from PDB structure are converted into ENM nodes) are set to the same fixed value, 1 amu, but actual atomic masses (`-mass`) were used in our ENM. The `-ca` flag creates an ENM model where amino acids' Cα atoms form nodes. In combination with `-ca`, the `-res` flag assigns the Cα nodes the whole residue mass. GENENMM Generates the interaction matrix for the ENM, written into `matrix.sdijf` file.
Next, the DIAGSTD function diagonalises the interaction matrix using small-block diagonalisation and iterative schemes (1).

DIAGSTD -i matrix.sdijf

where `-i` flag specifies input. The product of the diagonalisation is written into the `matrix.eigenfacs` file.
We calculate the inter-atom distance and cross-correlation of motion maps for the Cα ENM of the protein using SPACING and CROSCOR functions, respectively.

SPACING -pdb 6lu7_holo1.pdb -ca -het

and

CROSCOR -i matrix.eigenfacs -s 7 -e 31 -het

where `-s` and `-e` flags indicate the first and the last normal modes to include in calculation. Note, the first six normal mode frequencies, which correspond trivially to translational and rotational motion, are equal to zero.
Finally, fluctuation free energies for the set of the normal modes are calculated via the FREQEN function as follows

FREQEN -i matrix.eigenfacs -s 7 -e 106

The temperature (T) value used to calculate the partition function Z and Gibbs free energy G was 298 K, the default value for FREQEN function.

B.1. Fluctuation Free Energy Approximation. For convenience, DDPT estimates fluctuation free energy for each normal mode in dimensionless units $\frac{G}{k_B T}$ as follows

$$\frac{G}{k_B T} = -ln\left(\frac{1}{1-\exp\left(-\frac{\hbar\omega}{k_B T}\right)}\right) + \frac{1}{2}\frac{\hbar\omega}{k_B T}$$

Normal mode frequencies of global modes in proteins are in the acoustic regime (less than 10 THz) (2, 3). This fact is especially true for the slowest modes we investigated in this study. In this classical limit, $\frac{\hbar\omega}{k_B T} \ll 1$ at 298 K temperature. Therefore, FREQEN function in DDPT employs the following expression for fluctuation free energy calculation

$$\frac{G}{k_B T} \approx -ln\left(\frac{1}{1-\exp\left(-\frac{\hbar\omega}{k_B T}\right)}\right) \qquad [1]$$

In the differences (ΔG) and difference of a difference ($\Delta\Delta G$) in fluctuation free energy (**Eq. 2**) significance of $\frac{1}{2}\frac{\hbar\omega}{k_B T}$ term is negligible.

B.2. The N3 Inhibitor Coarse-graining. The N3 peptide-like inhibitor consists of four chemical constructs: 02J, AVL peptide, PJE and 010. DDPT reads each heavy atom from 02J, PJE and 010, and creates nodes with corresponding atomic coordinates and mass for each atom in elastic network.
This procedure is default for the `HETATM` record type in PDB files with the chosen GENENMM flags. However, the AVL peptide is classified as `ATOM` record type, thus it is treated in the same way as the amino acids in the main chain: the tripeptide is represented as three nodes with each amino acid's alpha carbon coordinates and mass corresponding to the amino acids' mass. In consequence, each ligand results in 32 elastic network nodes: 29 from 02J, PJE and 010 constructs, and 3 nodes from the AVL tripeptide - shown in figure S2.

C. Fluctuation Energy Convergence. The allosteric free energy change is calculated using the fluctuation free energy (**Eq. 6**) change for three forms of the protein:

$$\begin{aligned} \Delta\Delta G &= \Delta G_2 - \Delta G_1 \\ &= (G_{holo2} - G_{holo1}) - (G_{holo1} - G_{apo}) \\ &= G_{holo2} - 2G_{holo1} + G_{apo} \end{aligned} \quad [2]$$

For the apo form, the fluctuation free energy curve is smooth and stably converging with the number of modes included (**Fig. S4**). However, figure S5 shows a poorer convergence in the ratio of dissociation constants, due to the inherent noise-amplification in taking the difference of a difference in the (numerical) fluctuation free energies. However, the K_2/K_1 values fluctuate stably in the 1.02-1.06 range when the cut-off in mode number is between 23 and 33 modes. The higher-mode number structure that sets in beyond mode 50 is also apparent in the mutation scan calculations shown in S6. For this reason and three others stated in section 2 of the main paper text, we have chosen to stop summing at 25 non-trivial modes.

The 1-point mutational scan for the apo form is stable in respect of the identification of biologically active sites for increasing values of mode-sum cut-off (**Fig. S6A**). The same mutational scan, but for allosteric energy change, is less stable (for the same reasons of numerical-difference as in the sensitivity of K_2/K_1 of figure S5 above) (**Fig. S6B**). Nevertheless, the identified bioactive sites persist stably with mode cut-off. As the sign of the stiffening modelling local mutation is changed, we observed a similar, and induced, sign change in the allosteric response but, again, the patterns of bioactive residue persist. Visual inspection of the hydrophobic environment around residue 214 and 284-286 (**Fig. S7**) informed the choice of magnitudes of k_R/k in our ENM that corresponds to the experimental mutation.

The identified candidate regions that exhibit allosteric activity in our ENM study (**Tab. 1**) were visualised in 3D space (**Fig. 1**).

D. Definition of residues for dynamically allosteric control in SARS-CoV-2 M^{pro} ENM. We use results from the 1-point mutational apo scan (**Fig. 3A**) to suggest potential control residues on both homodimeric chains distant from the active sites. This map captures significant fluctuation free energy change in the ENM dynamics of residue 214 and 284-286 mutations. The active residue patterns converge with fluctuation mode summation. In order to be considered a candidate control residue, the residue must pass two criteria:

1. Absolute fluctuation free energy change must be greater or equal to the smallest absolute value of experimentally evidenced dynamically allosteric residues (214 and 284-286) at k_R/k=0.25 or 4.00.

2. Cα node distance between the candidate residue and the catalytic active site residues (H41 and C145) in the ENM must be greater or equal to twice the ENM cut-off value (16 Å).

The first criterion filters out all residues which do not contribute to free energy change significantly; the second criterion ensures that the control effect of mutation or binding at the candidate residue is not caused by spatial proximity. We made only one exception (for E14) which is located on the homodimer interface; this showed desirable activity but was 15.1 Å away from C145. Residues that passed the two criteria above are documented in table 1 and coloured black in figure 1 of the main manuscript text.

E. 2-point spring constant mutational scan. Double-mutation effects were explored from a continuous range of spring-stiffening and weakening in calculations of apo and allosteric free energies, restricted to the residue pair of C145 and H41, but with all possible pairwise combinations of spring constant change k_R/k in range from 0.25 to 4.00 over the first real 25 fluctuation modes. Results are displayed in (**Fig. S8**). We note the following preliminary findings:

- Region of wild-type values forms a curve that creates a larger parameter region for negative contributions to free energies in all apo scans.

- The allosteric scans indicate an approximately linear addition when the residue pairs are identical on the two chains, but when they differ generate a non-linearity in which stiffening at the C145 site has less effect than weakening.

- Although, apo scans for H41A-C145B and C145A-H41A are identical as expected, the corresponding allosteric scans.

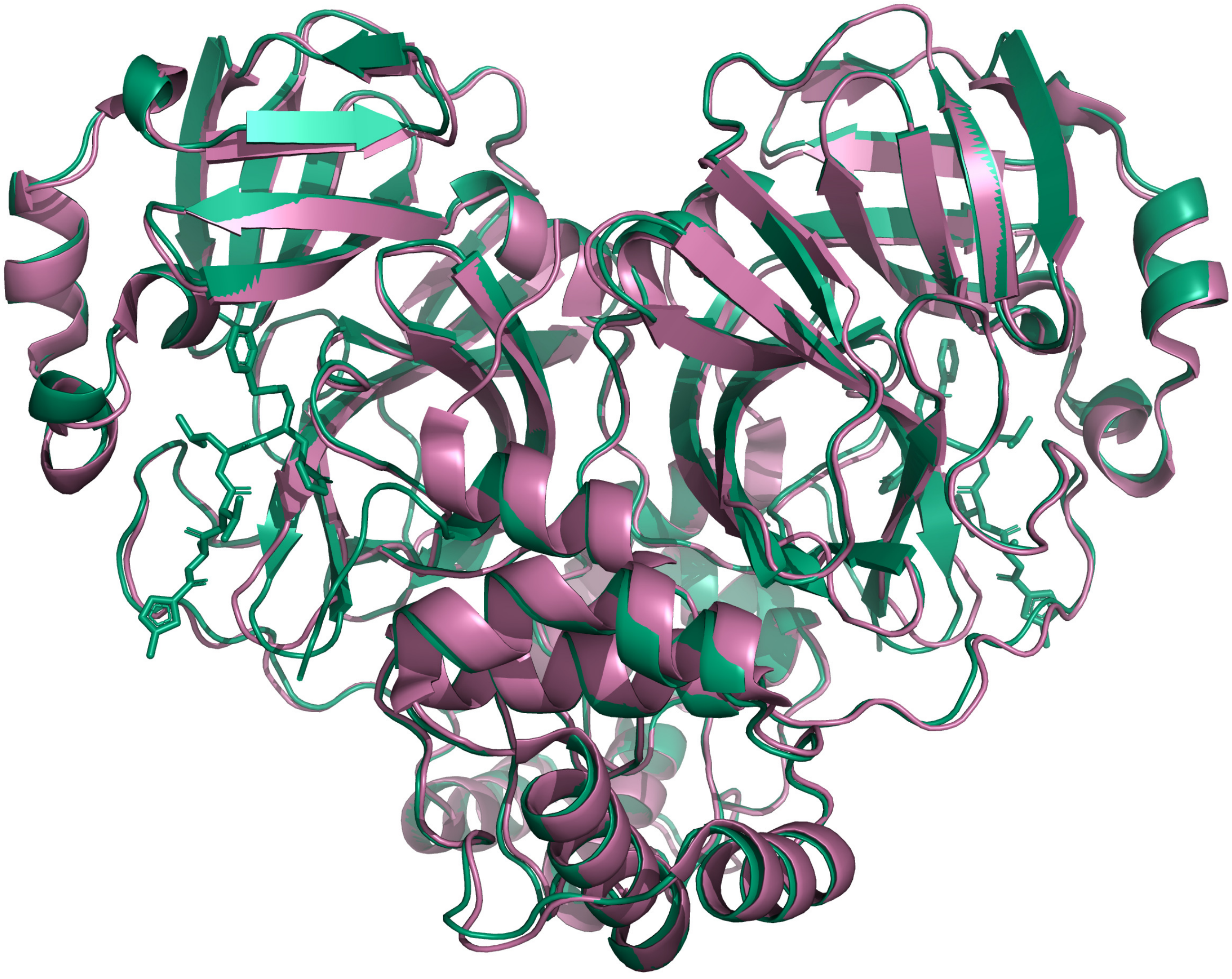

Fig. S1. Inhibited (green) and ligand-free (magenta) SARS-CoV-2 M^{pro} crystallographic structures superimposed using the Combinatorial Extension (CE) algorithm in PyMOL (Schrödinger). The RMSD between two structures is $1.48\,\text{Å}$. Both proteins are shown as secondary structure cartoons while N3 inhibitor is shown as sticks. PDB accessions are 6lu7 and 6y2e (4), respectively.

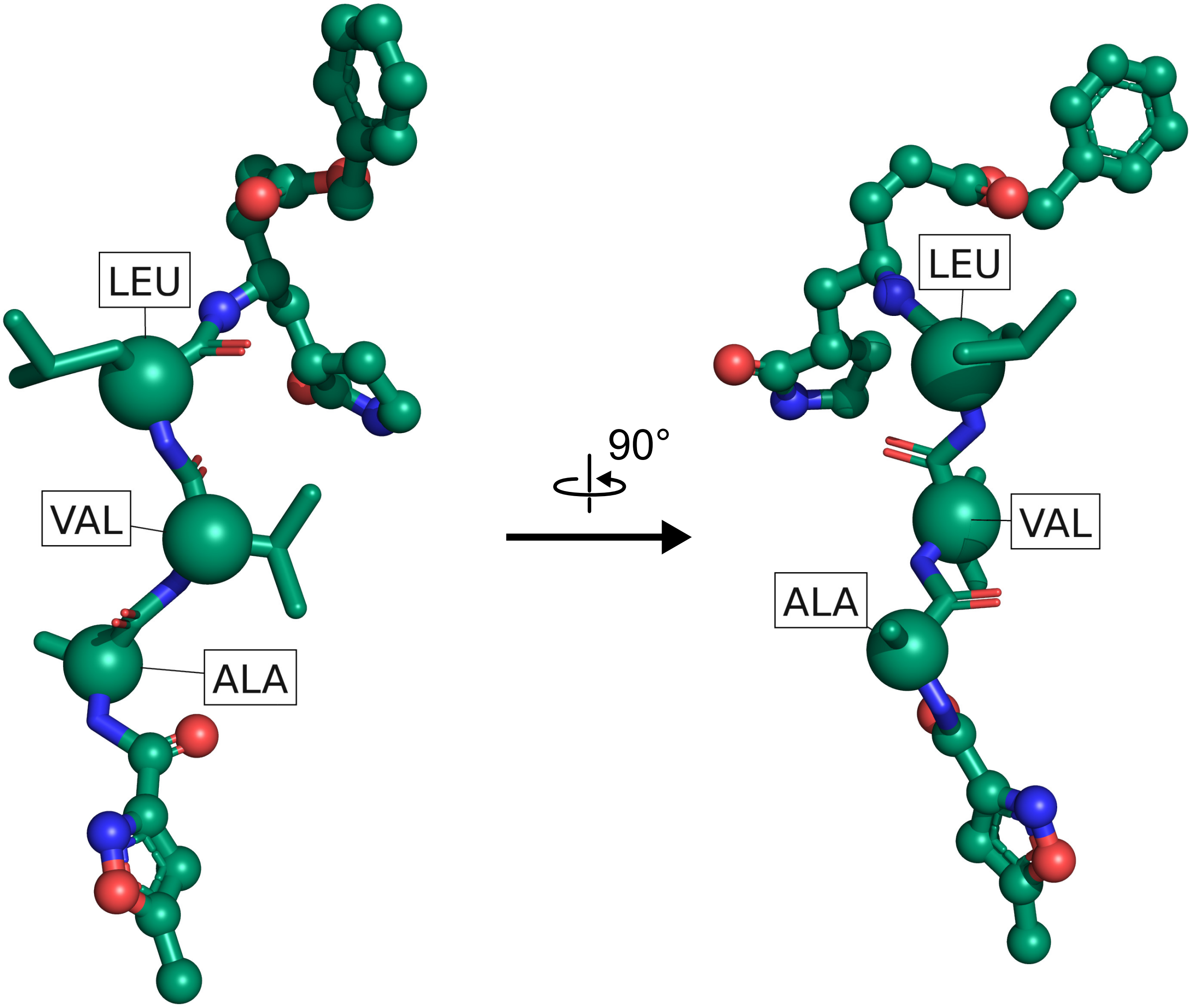

Fig. S2. Coarse-graining of the N3 inhibitor. The inhibitor is shown in stick representation while nodes produced in the coarse-graining are shown as spheres. C, N and O atoms are coloured green, blue and red. Three amino acids (alanine, valine and leucine) alpha carbon atoms are labelled with the three letter code. The spherical node size, i.e. volume, is normalised against C atomic mass.

.

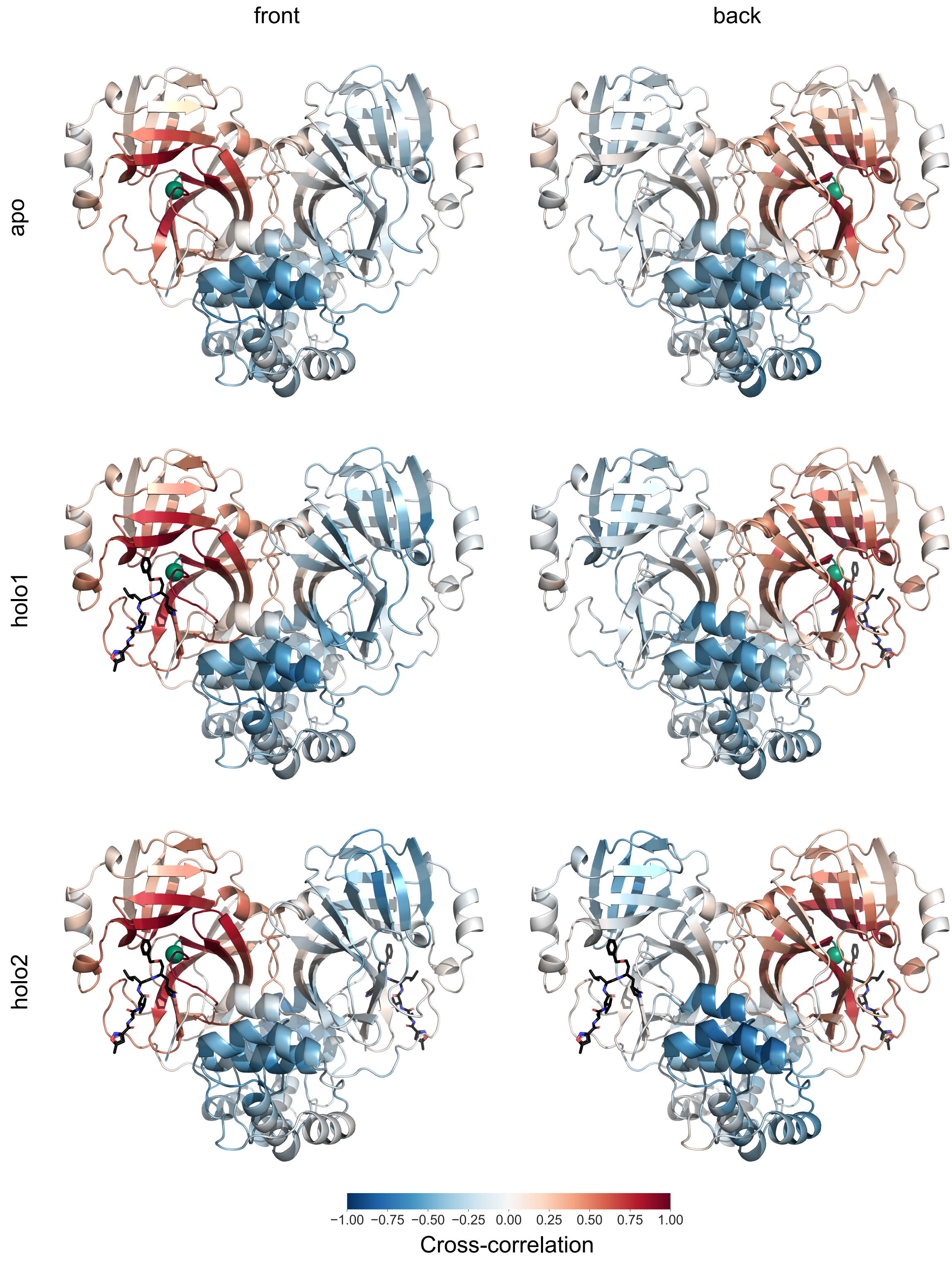

Fig. S3. A real-space representation of the main chain residue correlations in 6lu7 ENM shown in figure 3. Red indicates perfectly correlated (+1), blue is perfectly anti-correlated (-1), and white is uncorrelated motion (0), with respect to C145 on chain A (green sphere).

.

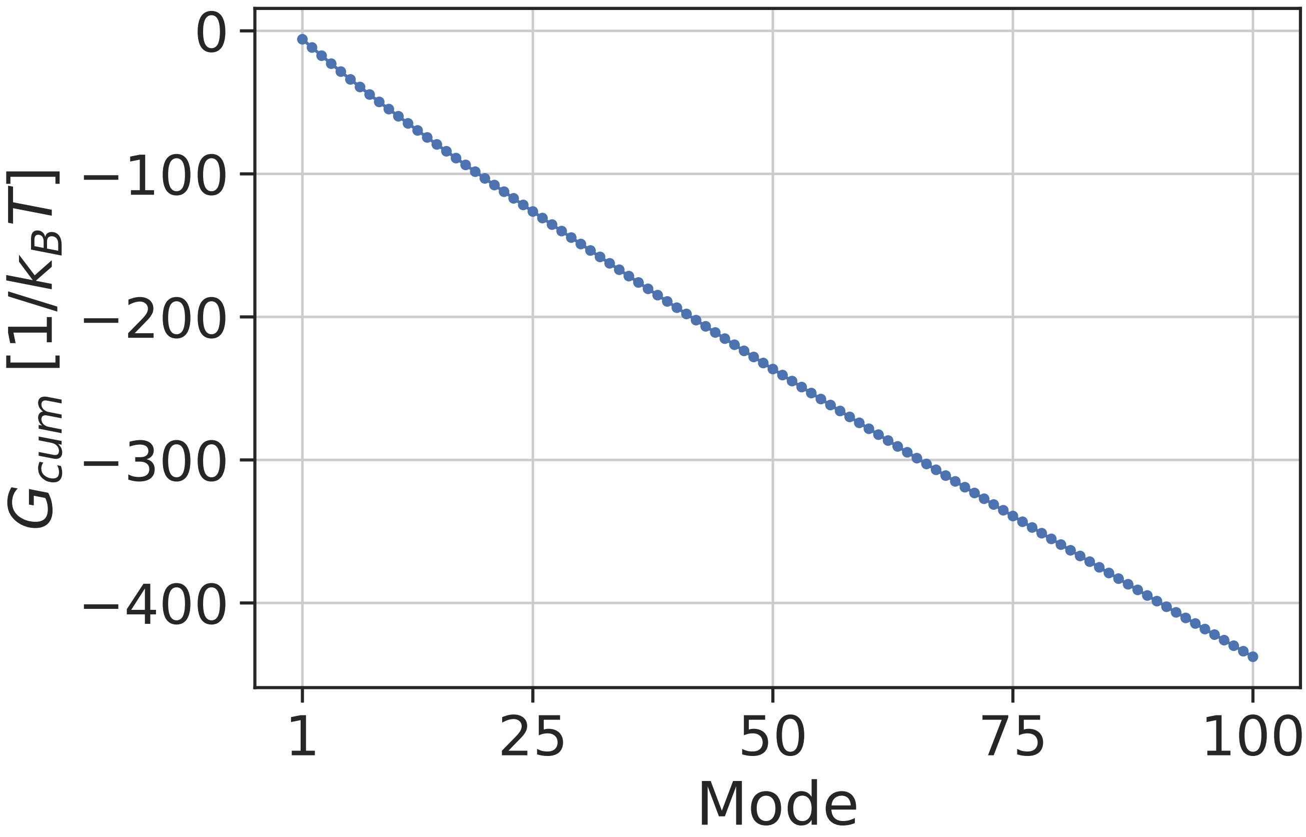

Fig. S4. Wild-type ligand-free SARS-CoV-2 M^{pro} ENM fluctuation free energy dependence on mode summation at $298\,\mathrm{K}$. All ENM Cα node masses correspond to amino acid mass, i.e. whole residue mass. Cut-off distance is 8 Å. All ENM spring constants are equal $1\,\mathrm{kcal}\,\mathrm{Å}^{-2}\,\mathrm{mol}^{-1}$

.

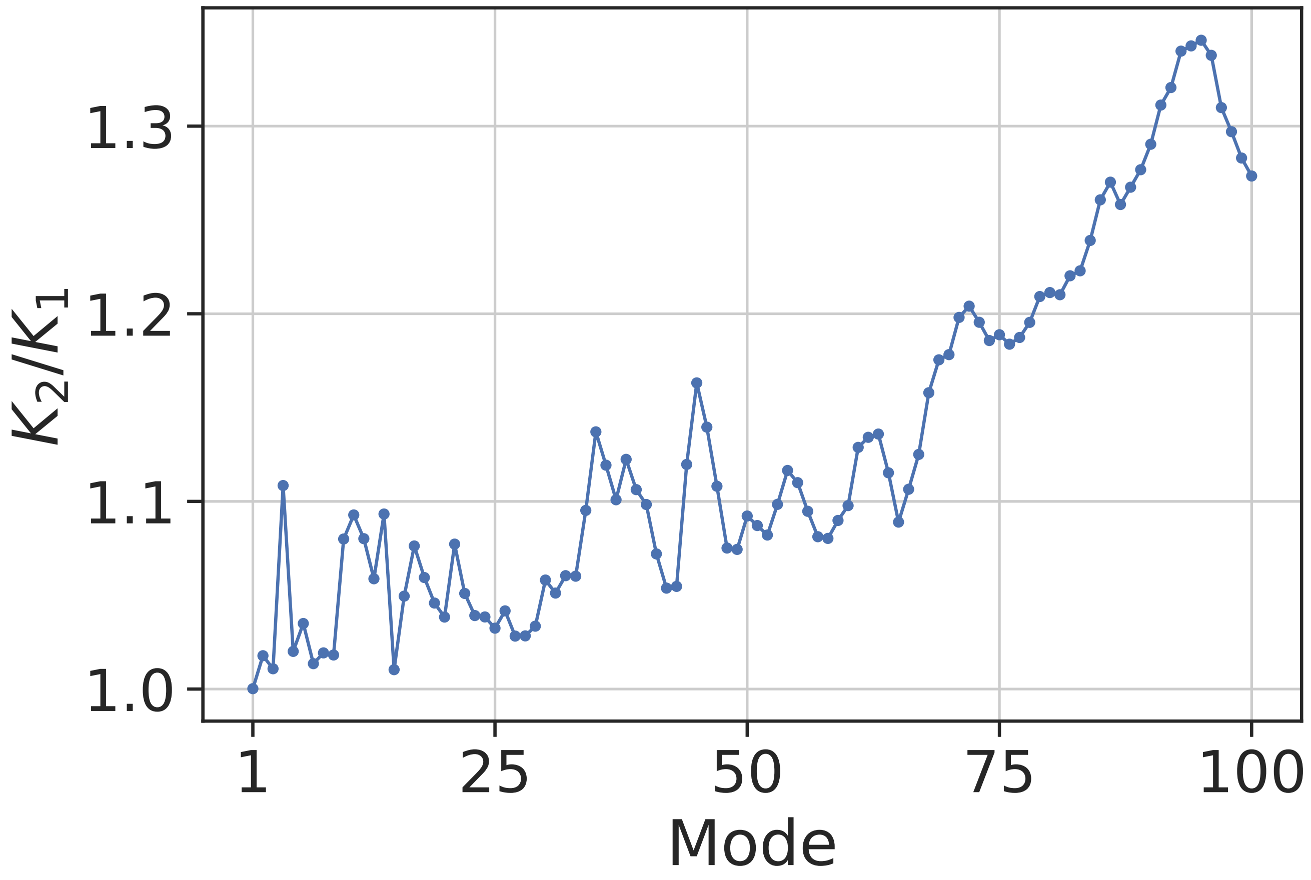

Fig. S5. Wild-type SARS-CoV-2 M^{pro} ENM cooperativity dependence on mode summation. All ENM node masses correspond to amino acid mass a node been derived from; while ligand nodes mass is assigned based on the element. Cut-off distance is 8 Å. All ENM spring constants are equal $1\,\mathrm{kcal}\,\mathrm{Å}^{-2}\,\mathrm{mol}^{-1}$.

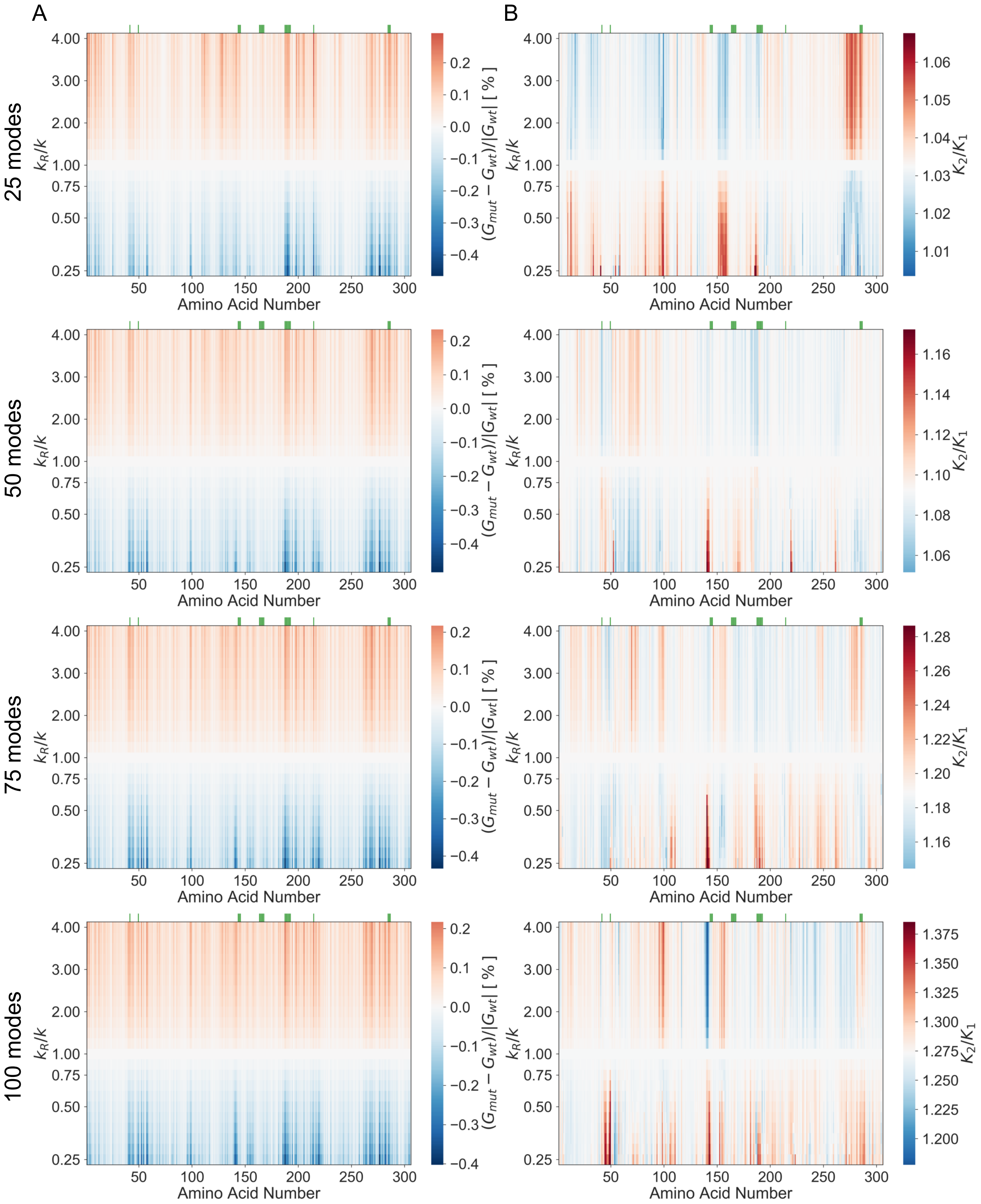

Fig. S6. 1-point mutational scan variation in M^{pro} (6lu7) ENM over 4 increasing cumulative mode values: 25, 50, 75 and 100 modes. (A) 1-point mutational scan of apo ENM. (B) A map for the global control space of allostery in M^{pro} calculated from the ENM.

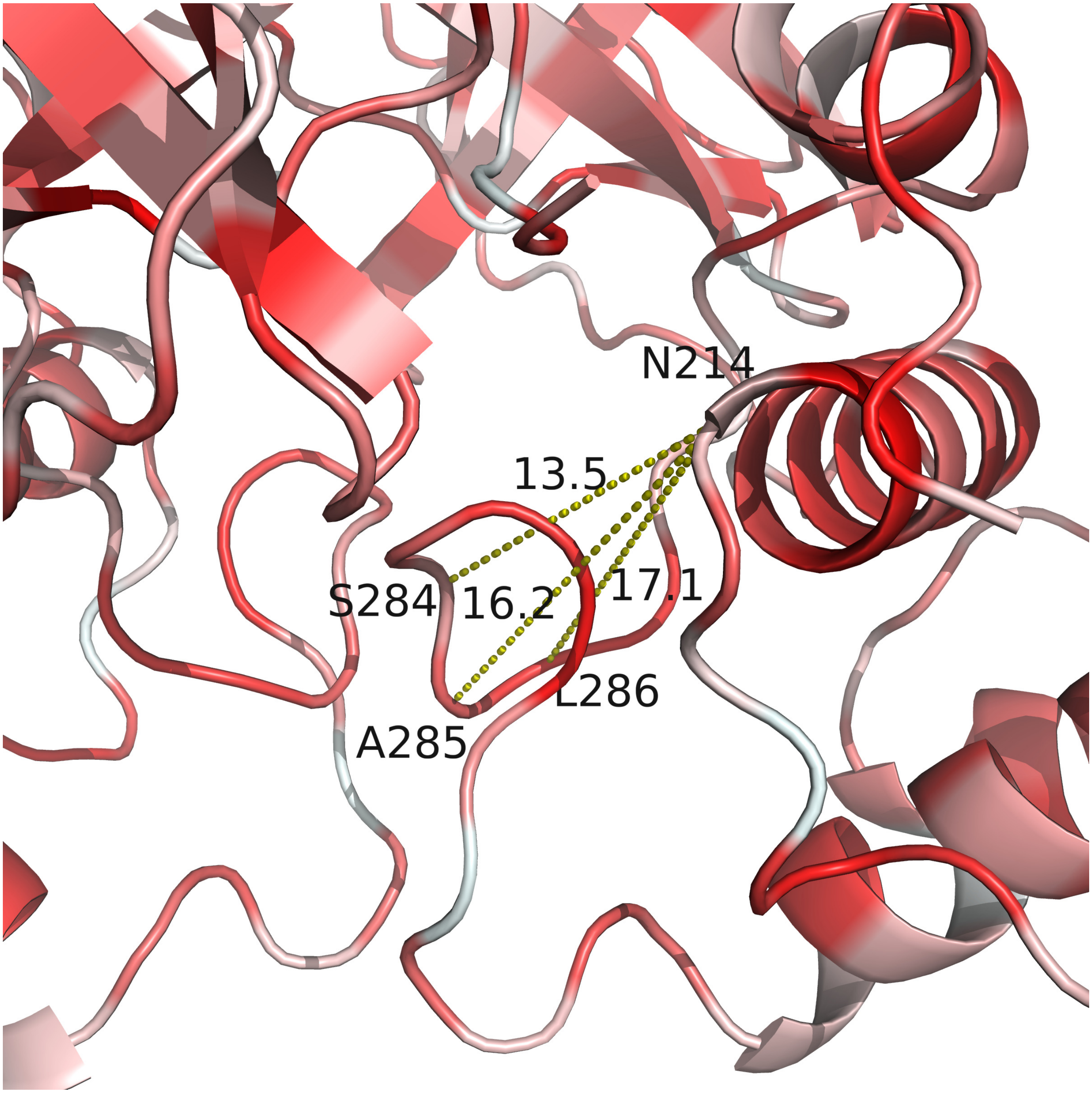

Fig. S7. Hydrophobic environment around residue 214 and 284-286 in SARS-CoV-2 M^{pro} based on Eisenberg amino acid hydrophobicity scale (5). Red illustrates hydrophobic residues while white shows less hydrophobic (hydrophilic) residues. Euclidean distance between N214 and S284-A285-L286 is shown with yellow dashed lines in Å.

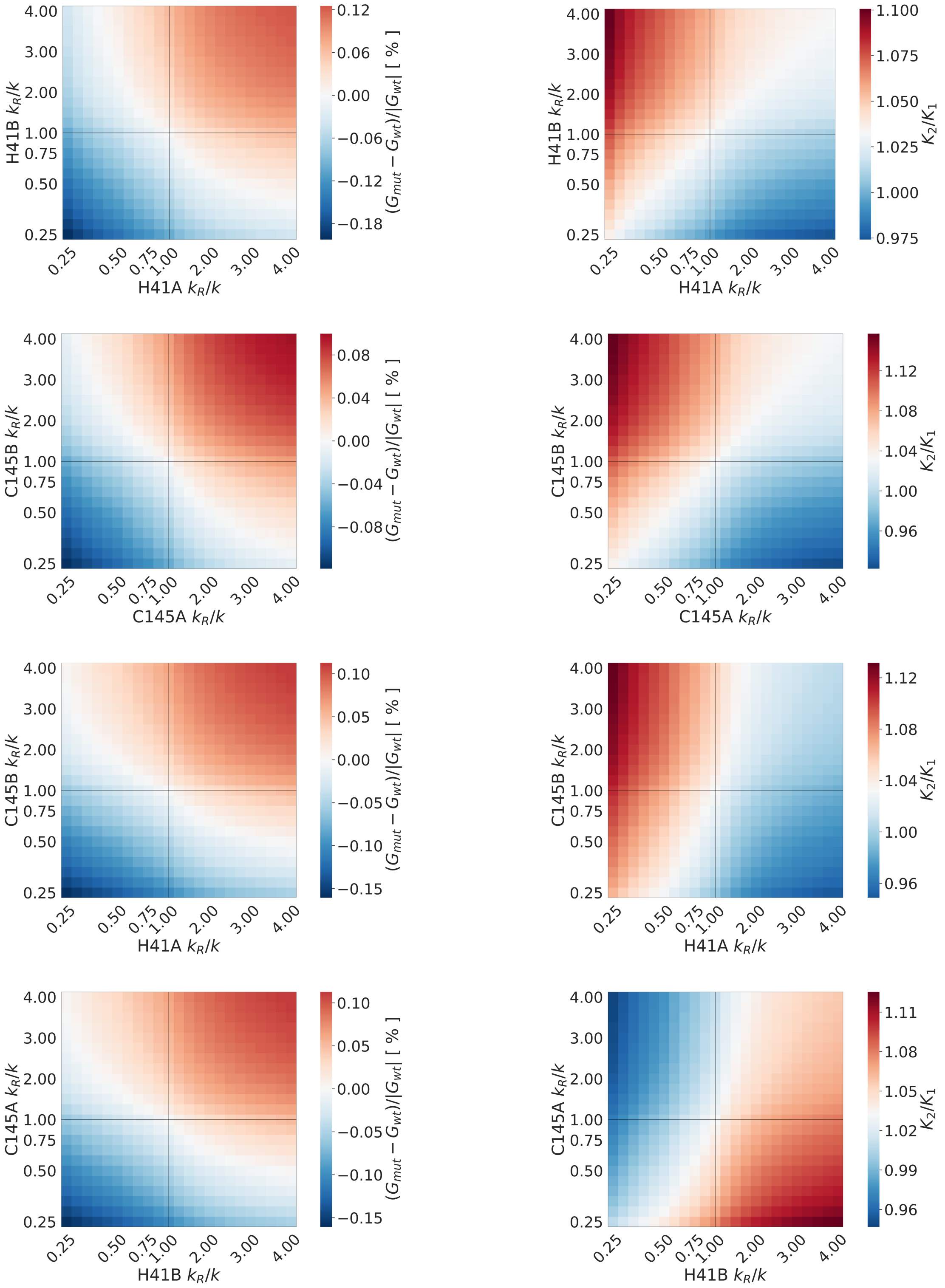

Fig. S8. 2-point mutational maps for 6lu7 ENM with two residue mutations, restricted to the pair C145 and H41, but with all possible pairwise combinations of spring constant change k_R/k in range from 0.25 to 4.00 over the first real 25 fluctuation modes. (*Left*) 2-point free energy mutation maps on apo structure. (*Right*) Maps for the 2D global control space of allostery in M^{pro}. Axis represent dimensionless change in ENM's spring constant (k_R/k) for two mutated residue with the single code amino acid name, number and chain ID shown, e.g. H41A refers to histidine 41 on chain A. Perpendicular lines to the axis span wild-type spring constant for each residue. The k_R/k range consists out of 23 values: 10 equally spaced values from 0.25 to 1.00, 12 equally spaced values from 1.00 to 4.00 and centre at $k_R/k = 1.00$.

References

1. TL Rodgers, et al., Ddpt: a comprehensive toolbox for the analysis of protein motion. *BMC Bioinforma.* **14**, 183 (2013).
2. JA McCammon, BR Gelin, M Karplus, PG WOLYNES, The hinge-bending mode in lysozyme. *Nature* **262**, 325–326 (1976).
3. A Nicolaï, P Delarue, P Senet, Theoretical insights into sub-terahertz acoustic vibrations of proteins measured in single-molecule experiments. *The journal physical chemistry letters* **7**, 5128–5136 (2016).
4. L Zhang, et al., Crystal structure of sars-cov-2 main protease provides a basis for design of improved α-ketoamide inhibitors. *Science* (2020).
5. D Eisenberg, E Schwarz, M Komarony, R Wall, Amino acid scale: Normalized consensus hydrophobicity scale. *J Mol Biol* **179**, 125–142 (1984).
